# A Novel Hyper-Heuristic Algorithm with Soft and Hard Constraints for Causal Discovery Using a Linear Structural Equation Model

**DOI:** 10.3390/e27010038

**Published:** 2025-01-06

**Authors:** Yinglong Dang, Xiaoguang Gao, Zidong Wang

**Affiliations:** School of Electronic and Information, Northwestern Polytechnical University, Xi’an 710129, China; dangyinglong@mail.nwpu.edu.cn (Y.D.); nwpu_wzd@mail.nwpu.edu.cn (Z.W.)

**Keywords:** causal discovery, structural constraint, structural equation model, hyper-heuristics

## Abstract

Artificial intelligence plays an indispensable role in improving productivity and promoting social development, and causal discovery is one of the extremely important research directions in this field. Acyclic directed graphs (DAGs) are the most commonly used tool in causal modeling because of their excellent interpretability and structural properties. However, in the face of insufficient data, the accuracy and efficiency of DAGs learning are greatly reduced, resulting in a false perception of causality. As intuitive expert knowledge, structural constraints control DAG learning by limiting the causal relationship between variables, which is expected to solve the above-mentioned problem. However, it is often impossible to build a DAG by relying on expert knowledge alone. To solve this problem, we propose the use of expert knowledge as a hard constraint and the structural prior gained via data learning as a soft constraint. In this paper, we propose a fitness-rate-rank-based multiarmed bandit (FRRMAB) hyper-heuristic that integrates soft and hard constraints into the DAG learning process. For a linear structural equation model (SEM), soft constraints are obtained via partial correlation analysis. The experimental results on different networks show that the proposed method has higher scalability and accuracy.

## 1. Introduction

The representation of causality relies on interpretable graph structures, and causal discovery learns these graph structures from data. One of the most classic and widely used examples is the DAG, which is widely used in various fields [1,2,3,4]. There are two common ways to build a DAG: building with expert knowledge and building with data learning. DAG construction with expert knowledge determines whether there is a causal link between nodes by consulting experts in related fields, using scoring and voting combined with the actual situation, to build the most consistent DAG. However, this approach to building DAGs on the basis of expert knowledge is time-consuming and expensive. Therefore, for large-scale DAGs, building suitable structures using only expert knowledge is unrealistic. Compared with expert knowledge methods, data learning methods are less resource-intensive and more efficient. However, because the size of the structure space of the DAG increases super-exponentially with the number of nodes and the number of states of the nodes, DAG learning becomes an NP-hard problem. To reduce the complexity of the computation and search in DAG learning, data learning and expert knowledge can be combined. Integrating expert knowledge into data learning and deleting unreasonable and unrealistic directed edges can ensure that the structure learned in the process of data learning is reasonable and legal. On the one hand, it can remove some variables that are not closely related to the model to be constructed in data processing, thus reducing the complexity of learning. On the other hand, when the amount of training data is insufficient or there is considerable noise, the learned DAG structure may differ greatly from the actual structure, and the expert can correct the learned structure to ensure that the constructed Bayesian network structure is in line with practical applications. In this paper, expert knowledge is used as a hard constraint in the DAG learning process.

Currently, the methods for learning DAGs from data fall into three main categories: constraint-based methods, score-based methods, and hybrid methods. The constraint-based method is divided into two steps: the first step is to conduct an exhaustive set of CI tests, and the second step is to infer a class of DAGs on the basis of the CI test. However, the DAG learning algorithms based on constraint satisfaction have two assumptions: causal faithfulness and causal sufficiency. The score-based method is also divided into two steps: score calculation, which involves the selection and score calculation of the candidate parent set of each node, and structure optimization, which uses a certain search strategy to find the optimal solution in the specified search space. The research on the first stage is mature, and scholars have made more improvements in the second stage. These algorithms can be divided into two categories: approximate methods and exact methods. The exact approach cannot solve the large-scale network learning problems, and the approximate learning algorithm focuses on improving the learning efficiency of the DAG and the maximum network size that can be learned, so it cannot guarantee that the learned result is the global optimal solution theoretically. Hill climbing (HC) [5], the most widely used approximate method, is prone to falling into local optima. To escape local optima, many bionic algorithms inspired by biological systems, such as the genetic algorithm (GA) [6], ant colony optimization (ACO) [7], particle swarm optimization (PSO) [8,9], artificial bee colony algorithm (ABC) [10], cuckoo optimization (CO) [11], firefly algorithm (FA) [12], bacterial foraging optimization (BFO) [13], and water cycle optimization (WCO) [14], have emerged in recent years. Although these optimization algorithms have demonstrated their accuracy and efficiency in DAG learning through experiments, the following challenges remain:

- The scalability of a single meta-heuristic algorithm needs to be improved.
- For a large DAG, a single meta-heuristic algorithm easily falls into local optima with low convergence accuracy.

Recently, hyper-heuristic algorithms have replaced meta-heuristic algorithms as the research hotspot in the field of combinatorial optimization and have been applied to many practical engineering problems, such as the knapsack problem (KP) [15], traveling salesperson problem (TSP) [16], and vehicle routing problem (VRP) [17]. While hyper-heuristic algorithms can be used to solve practical problems, many high-quality heuristic selection strategies are produced: choice function, FRRMAB, and several meta-heuristic strategies, such as simulating annealing (SA), tabu search, and swarm intelligent selection. In this paper, we employ FRRMAB as a hyper-heuristic high-level selection strategy.

In addition, some scholars have proposed hybrid methods, which use a method based on constraint satisfaction to limit the search space and then use a method based on score search to perform local optimization. The max–min hill climbing algorithm (MMHC) [18] is a classical hybrid algorithm for solving large-scale DAG learning problems. Limiting the search space can be considered a soft constraint, and the hybrid method is actually a learning method based on knowledge fusion [19]. In this paper, we use partial correlation analysis to identify the partial V-structure and limit the search space. These structure priors obtained through data mining are used as soft constraints to guide the search direction of the algorithm. In summary, in our algorithm, there are two kinds of structural constraints: soft constraints, which are applied by intervening in the learning process, and hard constraints, which force the learned network to meet certain conditions.

The main contributions of the paper are summarized as follows:

1. We use both soft and hard constraints to guide or limit the whole process of the hyper-heuristic algorithm.
2. We construct a low-level operator library of hyper-heuristic algorithms in the DAG learning field.
3. We propose a multi-population FRRMAB hyper-heuristic (M-FRRMAB-HH). Multi-population strategies can provide sufficient search space coverage and communicate with each other through migration operators through different groups.

This paper is organized as follows: Section 2 presents related works. Section 3 introduces the preliminaries. Section 4 describes our proposed algorithm. Section 5 discusses our experimental results. Section 6 concludes this paper.

## 2. Related Works

At present, the DAG learning problem is solved mainly as a combinatorial optimization problem or continuous optimization problem. For the combinatorial optimization problem, the core goal is to design a scoring function to evaluate the generated network and then find the network with the highest score. There are two main solutions: constraint satisfaction and score search. The methods based on constraint satisfaction can be divided into global structure learning or local structure learning.

Global structure learning attempts to discover the graph structure of all variables on the basis of the CI test. One of the classic algorithms is the SGS algorithm, which first removes the edges between the independent variables starting with the fully connected graph and then discovers the V structure and the orientation of the remaining undirected edges. The PC algorithm is an improvement over the SGS algorithm, reducing the time cost. However, the classical PC algorithm is related to the node order, so it can easily incorrectly remove the directed edge. A method called the PC-Stable [20] algorithm, which is independent of the node order, was subsequently proposed. Aiming at hidden variables and confounding factors, the FCI algorithm and its improved RFCI [21] method were proposed. Local structure learning focuses on identifying parent—child node sets or Markov blankets (MBs) in the DAG. The GS was the first algorithm used to discover the MB structure and consists of two steps: growth and reduction. The IAMB [22] algorithm and its improved versions, such as Inter-IAMB and Fast-IAMB, were subsequently proposed together. The MMPC, HITON-PC, and SI-HITON-PC [19] algorithms were also proposed for the discovery of parent—child node sets.

The methods based on score search can be divided into exact learning and approximate learning. Exact learning, which focuses on finding globally optimal structures for scoring, was first mentioned in node-ordered spaces because of its low complexity and ease of global searching. The idea of dynamic programming was first applied to DAG learning [23]. Yuan and Malone found that dynamic programming searches are very inefficient, so they used the A* [24] algorithm to search the order graph. The branch and bound (B&B) [25] algorithm was the first algorithm used to solve the global optimal structure in the DAG space. Since the superexponential number of states severely restricts the development of accurate learning in the DAG space, scholars have carried out a series of studies on how to prune the space without affecting global optimality. Integer linear programming (ILP) [26] is the best of these algorithms. It uses convex constraints and clival constraints to limit the search space. The structure learning problem is solved as an integer programming problem.

Unlike exact learning, approximate learning can be generalized to large DAGs. Approximate learning is the most widely used method. The search space can be divided into a DAG space, an equivalent class (EC) space and an ordering space. In the DAG space, various heuristic algorithms have been proposed to solve local optimum problems. In the EC space, greedy equivalence search (GES) [27] is the classic score search method; it includes two processes, forward search and reverse search; uses insertion and deletion operators, respectively, to achieve the neighborhood search of the DAG; and learns the final structure by constantly updating the optimal valence class. Ramsey et al. [28] developed a parallel version of GES, FGES, and Bernaola et al. [29] proposed the FGES-merge algorithm for hyperscale networks. The ordering space has the least complexity and is more efficient than the DAG space and the EC space. The K2 [30] algorithm was the first to explore how to obtain the optimal DAG corresponding to a given node order.

The continuous optimization problem is a new research direction that has emerged in recent years, but, once it was proposed, it was recognized by the majority of scholars. Its purpose is to construct the corresponding DAG for some multimodal data, such as parametric/nonparametric, linear/nonlinear, and Gaussian/nonGaussian data, and solve it using a continuous optimization algorithm. To characterize causality, such algorithms make assumptions about the distribution of the data in advance, such as the linear non-Gaussian cyclic model (LiNGAM) [31,32], postlinearity (PNL) [33], additive noise model (ANM) [34], and generalized linear model (GLM). On the premise that causal adequacy is satisfied, Shimizu [32] proved that the structure that best fits these hypotheses can be solved by independent component analysis. Zheng et al. [35] proposed a classical algorithm, NOTEARS, which represents a directed acyclic constraint with the use of a weight matrix and regards finding the optimal network as a process of loss minimization. In addition, many works based on NOTEARS, such as NOFEARS [36] and NOBEARS [37], have emerged in recent years. Yu et al. [38] proposed the use of a GNN to reconstruct the loss function. Then, autoencoders [39] and reinforcement learning [40] were also introduced to perform as solvers.

To date, approximate learning has been the most widely used method in practice. However, their optimization strategies have obvious randomness, and the complexity of their search space increases exponentially when the scale of the variables increases, so the search accuracy of random strategies is difficult to guarantee, and expert knowledge should be introduced to guide the direction of greedy search to rescue. However, the current approximate algorithms [6,7,8,9,10,11,12] cannot glean knowledge from data without given constraints nor do they investigate how greedy optimization can be performed heuristically under given structural constraints. Therefore, integrating expert knowledge and structural priors into the heuristic search process is a topic worth studying.

## 3. Background

### 3.1. DAG Model

*G* represents a DAG, which can be represented as a binary group G=(V,E), where $V=\left\{X_{1},\cdots ,X_{n}\right\}$ represents the set of variables (or the set of nodes), and *E* represents the set of directed edges between variables.

### 3.2. SEMs

SEM is usually used to represent the DAG structure under continuous variables:

(1) $$x_{i}=f_{X_{i}}\left(pa\left(X_{i}\right),u_{X_{i}}\right)$$

where the functions $f_{X_{i}}$ are the decision function, $pa\left(X_{i}\right)$ represents the parent node set, and $u_{X_{i}}$ represents the noise distribution. In particular, the decision function can be defined as either linear or nonlinear. When it is defined as linear, we can define a linear SEM:

(2) $$x_{i}=w_{X_{i}}^{T}pa\left(X_{i}\right)+u_{X_{i}}$$

### 3.3. Scoring Function

For linear SEM, we use the BIC score, which is defined as follows:

(3) $${Score}_{BIC}=\sum_{j=1}^{n}\left(NLL\left(X_{j},pa\left(X_{j}\right),{\hat{\theta }}_{j}^{mle}\right)+\frac{\left|{\hat{\theta }}_{j}^{mle}\right|}{2}\log m\right)$$

where $NLL\left(X_{j},pa\left(X_{j}\right),{\hat{\theta }}^{mle}\right)$ represents the negative log-likelihood, and the calculation formula is as follows:

(4) $$NLL\left(X_{j},pa\left(X_{j}\right),{\hat{\theta }}_{j}^{mle}\right)=\sum_{j=1}^{m}{\left(x_{ij}-{\left({\hat{\theta }}_{j}^{mle}\right)}^{T}pa\left(x_{ij}\right)\right)}^{2}$$

(5) $${\hat{\theta }}_{j}^{mle}={\left(x^{\prime }x\right)}^{-1}x^{\prime }x_{j}$$

where $x_{i}$ denotes the m×1 vector of observations on $X_{i}$, in which the *j*th element of $x_{i}$ is $x_{ji}$.

## 4. Methodology

In our algorithm, structural constraints consist of two parts: soft constraints and hard constraints. For soft constraints, we first mine conditional independent information according to the SPPC [41] algorithm, identify the partial V-structure, obtain three restricted search spaces (GSS, CSS, and LSS), and integrate these structural priors into the search process of the hyper-heuristic algorithm as soft constraints. For hard constraints, we randomly select a certain percentage of correct edges and a certain percentage of forbidden edges as expert knowledge to enforce in the final generated structure. In addition, the three search spaces described above, CSS, GSS, and LSS, are also forced to remove those edges that were determined from expert knowledge to exist or not exist, forming three new, narrower search spaces.

### 4.1. Proposed M-FRRMAB-HH Algorithm

**Figure 1:**
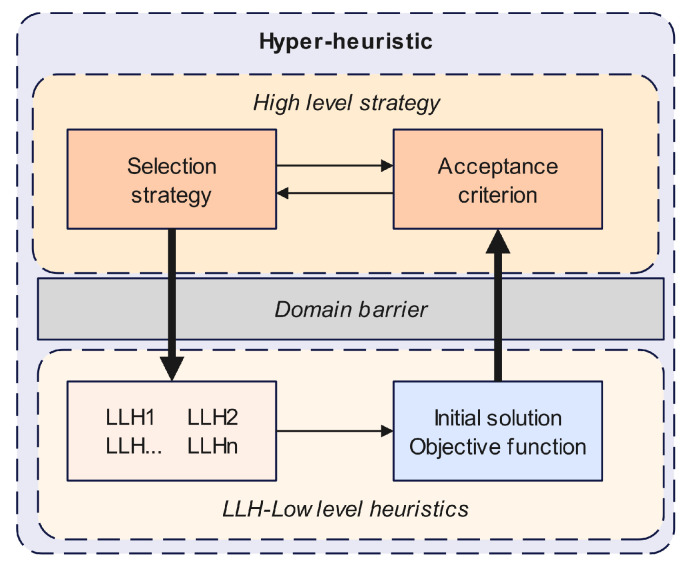
Proposed framework of the hyper-heuristic.

Figure 1 shows the framework of the proposed hyper-heuristic solver, which consists of two levels: the low-level heuristics (LLHs) and the high-level strategy. In the low-level problem domain, we define a set of scheduling heuristic operators with the corresponding expertise, while, in the high-level control domain, we design appropriate selection strategies and acceptance criteria to select high-quality heuristic operators and maintain the diversity of choices.

### 4.2. The High-Level Strategy

In this paper, FRRMAB is used as a high-level selection strategy, and Accept All Moves is used as an acceptance criterion. It has the following three main features:

1. A sliding window is used to store the performance information of the recent operator.
2. A decay mechanism is used to increase the selection frequency of the operator with good performance.
3. Each subgroup executes its own independent high-level policy and communicates when the interaction conditions are met.

Our high-level selection strategy has two main parts: credit assignment and operator selection. The most critical part of credit assignment is the metric that measures the performance of the operator. In DAG learning, the most commonly used index for measuring the performance of an operator is the improved value of the BIC score. However, improvements in BIC scores are much larger and easier in the early stages than in the later stages, so we used fitness improvement rates (FIRs) as an assessment measure [42] and converted them linearly into intervals (0.1, 0.5).

(6) $${FIR}_{i,t}=\frac{{pf}_{i,t}-{cf}_{i,t}}{-1\times {pf}_{i,t}}$$

where ${pf}_{i,t}$ represents the score of the parent; ${cf}_{i,t}$ represents the score of the child.

The sliding window, whose length is W, is divided into two lines: the first line records the serial number of each selected operator, and the second line records the FIR after the operator is executed. When the algorithm is running, the most recently selected operator index number and corresponding FIR are added to the end of the sliding window, while the first record is deleted to keep the window size unchanged. To increase the likelihood of choosing the best-performing operators, we use a decay mechanism for credit assignment. This process is as follows:

1. We calculate the sum of the FIRs for each operator in the second row, denoted as ${Reward}_{i}$.
2. ${Reward}_{i}$ is sorted in descending order and assigned a ranking. The first place has a rank of 1, and so on.
3. ${Reward}_{i}$ is converted to ${Decay}_{i}$ according to the following formula:
  (7) $${Decay}_{i}=D^{{Rank}_{i}}\times {Reward}_{i}$$
  where D represents the decaying factor, and its value is (0, 1).
4. The credit assignment value for each operator is calculated as follows:

(8) $${FRR}_{i,t}=\frac{{Decay}_{i}}{\sum_{j=1}^{K}\left({Decay}_{j}\right)}$$

The pseudocode of credit assignment is given in Algorithm 1. After the credit assignment is complete, we use a bandit-based operator selection scheme to select the operator, which uses the upper confidence bound (UCB) algorithm. The UCB algorithm assigns a value to each operator via the following formula:

(9) $${FRR}_{i,t}+\sqrt{\frac{2\times \ln\sum_{j=1}^{K}n_{j,t}}{n_{i,t}}}$$

where $n_{i}$ represents the number of times that the underlying operator i is selected by the high-level strategy. At the beginning of the search, we randomly select operators until no operator has been applied at least once and then use FRRMAB to select the operator with the largest value.
**Algorithm 1:** Procedure for the Credit Assignment.
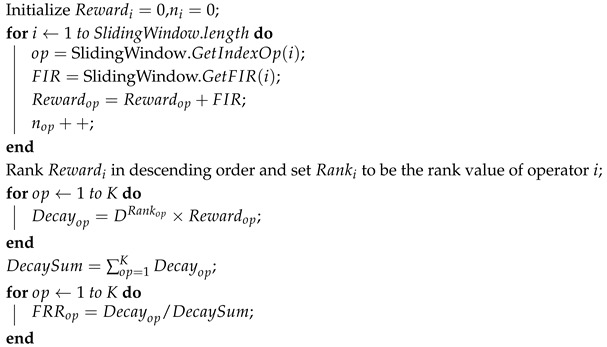


### 4.3. Low-Level Heuristics

The LLH pool is an important part of the hyper-heuristic algorithm, which is as important to the whole algorithm as the foundation of a tall building. Experimental studies have shown that the design of LLHs has a great effect on the final efficiency of the hyper-heuristic algorithm [43]. The design principle of LLHs is simple and rich. Operators require simple design: because LLHs are too complex, the final algorithm generated by the high-level heuristic strategy is too complex and consumes too much unnecessary operation time. Operators require a rich design, because only diversified operators can make the search of various problem domains richer, better, and faster to find the optimal solution. Various optimization algorithms and their improvements can be put into LLHs. For the DAG learning domain, 13 operators are extracted to construct the underlying operation base by analyzing various heuristic algorithms. Below, we introduce the source and working principle of each operator in detail.

#### 4.3.1. Mutation Operator and Neighborhood Perturbation Operator

The mutation operator is derived from the BNC-PSO algorithm, and the modification we made is to limit the search space to the GSS. The neighborhood perturbation operator randomly selects an edge to add, remove, or reverse to ensure that these edges are within our limited search space (LSS).

#### 4.3.2. Cognitive Personal Operator

The cognitive personal operator is derived from the BNC-PSO algorithm. In this operator, if the generated random number is less than the learning probability *p*, each individual learns from its historically optimal structure; otherwise, the individual remains unchanged. The learning probability *p* varies linearly from 0.82 to 0.32 with the number of iterations.

#### 4.3.3. Cooperative Global Operator

The cooperative global operator is also derived from the BNC-PSO algorithm. In this operator, if the generated random number is less than the learning probability *p*, each individual learns from the globally optimal structure; otherwise, the individual remains unchanged. The learning probability *p* varies linearly from 0.1 to 0.5 with the number of iterations.

#### 4.3.4. Chemotactic Operator

The chemotactic operator is derived from the BFO algorithm. To improve the efficiency of large-scale DAG learning, only one node is operated on at a time. That is, the addition action is greedy to add the parent node if the score increases, the delete action is greedy to remove the existing parent nodes if the score increases, and the reverse action is greedy to reverse the parent node if the score increases. These actions work only in the GSS.

#### 4.3.5. Elimination and Dispersal Operator

The elimination and dispersal operator is derived from the BFO algorithm, which restarts the parent set of only one node of the globally optimal structure. The procedure is as follows:

1. Select a node *X* in a preset order and delete all the parent nodes of this node.
2. Parent nodes are added to *X* if the score increases, and the resulting set of parent nodes is then sorted by partial correlation values as the potential parent importance of *X*. Then, all the parent nodes of X are deleted.
3. Add nodes from the potential parent to *X* one by one. If the score increases, both the node and its parent are added as the parent of *X*, while the new structure is used as the new starting point.
4. Greedily remove the nodes that are currently least able to increase the score and use the new structure as a new starting point until the score cannot be increased by removing the parent node.
5. Greedily invert the parent of X if the score can be increased.

The elimination and dispersal operator starts when the generated random number is less than the parameter, and the parameter is dynamically adjusted according to the following formula:

(10) $$c_{3}=0.1+0.9\left(L/L_{\max}\right)$$

where *L* represents the number of iterations in which the highest score does not increase, and  $L_{\max}$ represents the maximum number of allowed iterations in which the highest score does not increase.

#### 4.3.6. Employed Bees and Onlooker Bees

Both the employed bees and onlooker bees are derived from the ABC algorithm. The employed bees perform a local mountain climbing operation on the individual itself. The onlooker bees use roulette to select a good individual for climbing operations. Both of these are local optimization operators that operate within a restricted search space, GSS.

#### 4.3.7. Employed Bees and Onlooker Bees

The scout bees are derived from the ABC algorithm. To improve the learning efficiency of large DAGs, we consider restarting the parent set of only one node of the globally optimal structure. The procedure is as follows:

1. Select a node *X* in a preset order and record its parent set as *P*.
2. Pick one node in *P* and invert the edge.
3. Execute the addition, deletion, and reverse chemotaxis operators in sequence. If the score increases, the new DAG replaces the original DAG. Continue with Step 2 until *P* is empty.

When the iteration where the highest score is not increased is greater than $l_{m}$, the scout bee can perform; otherwise, it remains unchanged.

#### 4.3.8. Moth–Flame Optimization

Moth–flame optimization is based on a moth–flame optimization algorithm that randomly arranges the historically optimal structures of individuals as flames. The moth flying around the flame refers to the moth learning from its flame, and its learning method is similar to that of the BNC-PSO algorithm.

#### 4.3.9. Teaching-Learning-Based Optimization

In the teaching-learning-based optimization operator, each student is randomly assigned a collaborator and learns from the collaborator if the score is higher than their own, and its learning method is similar to that of the BNC-PSO algorithm.

#### 4.3.10. Expert Knowledge Operator

The expert knowledge operator selects a certain proportion of individuals to accept structural constraints, that is, to include all hard constraints and V structures.

#### 4.3.11. Pruning Operator

The pruning operator is designed to avoid overfitting by setting a threshold μ and then removing all edges that cause the score to increase less than μ.

### 4.4. Framework of Our Algorithm

The pseudocode of the M-FRRMAB-HH algorithm we propose is shown in Algorithm 2. First, the initial population contains the edges determined by the hard and soft constraints, and it is evenly divided into several subgroups, each of which performs the high-level selection strategy independently. When the migration condition is satisfied, information is exchanged between subgroups. In addition, when the score cannot be improved in the GSS, the search space switching operator is executed to convert the GSS to a CSS. Finally, the algorithm stops when any termination condition is satisfied.

#### 4.4.1. Migration Operator

The migration operation records the highest score structure of each subgroup, swaps the highest and lowest score structures, and is controlled by two indicators: the minimum number of interval iterations and the inbreeding rate. In this paper, the minimum number of interval iterations is set to a smaller value of both 100 and *N*. The inbreeding rate, which prevents premature population maturation, is designed as the number of close relatives of the highest score structure of each subgroup and the global optimal structure divided by the number of subgroups and was limited to no more than 0.6 in this study. In this study, if the Hamming distance between the highest score structure in the subgroup and the global optimal structure was less than 4, we considered them to be close relatives.
**Algorithm 2:** M-FRRMAB-HH.
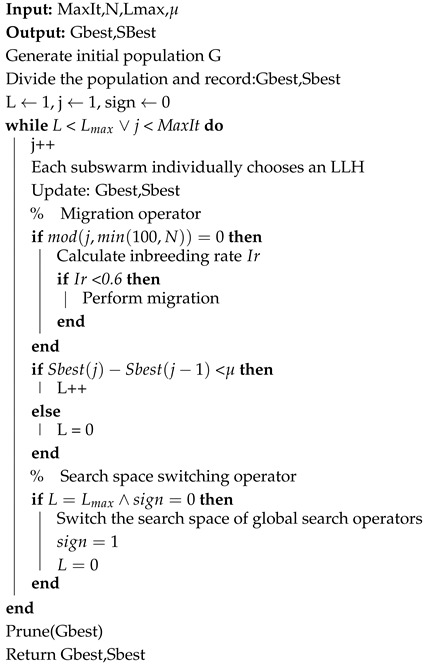


#### 4.4.2. Search Space Switching Operator

When the DAG is large or complex, the use of the GSS as the search space is prone to the incorrect deletion of some correct edges. Therefore, we considered introducing a search space switching operator to further improve accuracy, which is executed to correct possible errors due to an incomplete search space in the GSS when the number of iterations without increasing the global maximum score reaches $L_{\max}$. After the search space is switched, the scout bees as well as elimination and dispersal operators work within the CSS. Obviously, working on the GSS can improve the efficiency of our algorithm early on, and switching to the CSS can further improve the accuracy of our algorithm later on.

## 5. Experiments

Several algorithms and benchmark networks were selected to evaluate the performance of our proposed M-FRRMAB-HH algorithm. All the experiments were performed on an AMD 1.7 GHz CPU with 16 GB of RAM.

### 5.1. Networks and Datasets

In our experiment, all datasets were generated through six benchmark networks from the BNLEARN repository (https://www.bnlearn.com/bnrepository/, accessed on 9 October 2024), which are summarized in Table 1. Of the six networks, Alarm [44] belongs to the medium network. Hepar2 [45] and Win95pts are large networks. Andes [46], Munin [47], and Pigs are very large networks.

For linear SEM, we designed three SEMs to generate data, including Gaussian and non-Gaussian data. For each combination of network and SEM, we randomly generated a dataset of 1000 samples.

(11) $$\begin{array}{c} \left(1\right)x_{i}=w_{1X_{i}}^{T}pa\left(X_{i}\right)+N\left(0,1\right)\\ \left(2\right)x_{i}=w_{2X_{i}}^{T}pa\left(X_{i}\right)+N\left(0,1\right)\\ \left(3\right)x_{i}=w_{1X_{i}}^{T}pa\left(X_{i}\right)+\mathrm{rand}\left(-1,1\right) \end{array}$$

where $w_{1X_{i}}=\pm 1+N\left(0,1\right)/4$, and $w_{2X_{i}}=\mathrm{rand}\left(0.2,1\right).$ SEM (1) and SEM (2), since their noise follows a Gaussian distribution, belong to the Gaussian model. For SEM (3), the noise is uniformly distributed and is a non-Gaussian model.

We used different metrics to evaluate the effectiveness and efficiency of the algorithm, which are as follows:

- BIC: BIC score of the learned network (higher is better).
- SBS: BIC score of the original network.
- AE: The number of edges not present in the original network but added.
- DE: The number of edges present in the original network but undetected.
- RE: The number of edges correctly detected but in the opposite direction.
- RT: Running time of the algorithm.
- F1: F1 score of the learned network (higher is better).

### 5.2. Comparison Algorithms and Parameter Settings

We compared the M-FRRMAB-HH algorithm with five common algorithms, including the PC-stable, LiNGAM, PCS [48], BNC-PSO, and NOTEARS algorithms (https://github.com/xunzheng/notears, accessed on 9 October 2024). NOTEARS was tested in its Python implementations, and the other algorithms were run in MATLAB R2020a.

The main parameters of M-FRRMAB-HH are listed in Table 2. Since the causal relationship between variables is easier to negate than to establish, we set the constraint rate *p* for an edge that was determined to exist to 0.1 and the constraint rate *q* for an edge that was determined to not exist to 0.5. The parameters of the other algorithms were set according to the original literature, but soft constraints were introduced in the BNC-PSO algorithm to enable a fair comparison with our proposed algorithm in terms of global search performance.

### 5.3. Performance Evaluation of M-FRRMAB-HH

To evaluate the performance of M-FRRMAB-HH, we list the means and standard deviations of 10 runs of the algorithm on each dataset in Table 3.

First, for all the datasets on the three SEMs, the BIC scores of the learned network meet or exceed the BIC scores of the original network, and the standard deviation of both the BIC scores and F1 scores is 0. This shows that for different SEMs, our algorithm can learn a high-score network with stable F1 scores, which means that our proposed algorithm can work stably for both Gaussian and non-Gaussian models. For structural errors on the Alarm and Hepar2 networks, the AE, DE, and RE on all datasets are zero, indicating that our algorithm can stably learn a DAG that is consistent with the original network on the two networks. For the Win95pts and Andes networks, only on SEM2 is an edge incorrectly identified. For the Munin and Pigs networks, the networks learned on all datasets are missing 53 or 324 edges, with a small number of added edges and misdirected edges on some datasets. Notably, for all the datasets with structural errors, the BIC scores are higher than the SBS scores. After analysis, these false edges occurred because the dataset does not support them. By looking at all of these structural errors and BIC scores, we believe that our proposed algorithm can stably learn a high-scoring structure with a score no lower than that of the original network. On the basis of the above observations and analysis, we can conclude that our proposed algorithm is an effective and reliable method for learning the DAG on a linear SEM.

To illustrate the effect of expert knowledge on the performance of our proposed algorithm, we report the algorithm’s performance without hard constraints (p = 0, q = 0). As shown in Table 4, all datasets in the four networks Alarm, Hepar2, Win95pts, and Andes, regardless of expert knowledge, output a structure with the same BIC score and F1 score. For the Munin and Pigs networks, after removing the constraint of expert knowledge, the output network has a higher BIC score, but the number of DEs also increases. This is because expert knowledge contains some of the real edges that can cause the BIC scores to decrease. For run time, after removing the constraint of expert knowledge, the run time increases on all datasets, and the increase is particularly significant on the large Andes and Pigs networks. In summary, the constraint of expert knowledge can reduce the time cost of the algorithm and can correct the structural errors in the output structure.

LLHs contain several operators, among which the restart operator (elimination and dispersal operator, scout bees) is the core operator that hops out of the local optimum and the operator with the highest time cost. Therefore, in our algorithm, these two operators are likely the operators that have the greatest impact on the efficiency of the algorithm. To explore the working mechanism and function of the restart operator in the algorithm, we generated three new LLHs by eliminating the restart operator: LLH1 (remove elimination and dispersal operator from LLH), LLH2 (remove scout bees from LLH), and LLH3 (remove two restart operators from LLH). Table 5 shows the performance of our algorithm under the three new LLH pools.

Comparing Table 3 and Table 5, we can see that for the Alarm and Pigs networks, all three derived algorithms can output the same structure of BIC scores and F1 scores as the original algorithm. For the other four networks, the graph accuracy of the structure output by the algorithm under LLH3 decreases and the output structure is no longer stable. This shows the necessity of the restart operator. For the running time, the running time on LLH3 is significantly shorter than that on the other LLHs, which indicates that the addition of restart operators increases the time cost of the algorithm. Comparing LLH1 and LLH2, we find that for both the Hepar2 and Win95pts networks, neither of the derived algorithms can output a stable structure. This shows that the two cannot replace each other and can work better if they are used together. On the basis of the above analysis, we can conclude that both restart operators play important roles in running the algorithm and that removing either operator reduces the performance of the algorithm.

### 5.4. Comparison with Some Other Algorithms

To compare the graph accuracy between the networks learned by different algorithms, we report the F1 scores and BIC scores on different SEMs and networks in Table 6 and Table 7, with a sample size of 1000 and the best results on each dataset highlighted in black. To make a fair comparison, our algorithm did not take into account expert knowledge.

The M-FRRMAB-HH algorithm achieved the highest BIC score on 15/18 datasets and the highest F1 score on all datasets. These high F1 scores and low BIC scores occurred because the data were not faithful to the original structure. The comparisons of the BIC scores supported the conclusion that M-FRRMAB-HH > NOTEARS > BNC-PSO > PCS > LiNGAM > PC, and the ranking of the F1 scores was M-FRRMAB-HH > BNC-PSO > NOTEARS > PCS > PC > LiNGAM. Therefore, we believe that compared with the other five algorithms, our proposed algorithm has higher accuracy.

For NOTEARS, there were 11 outputs on the 18 datasets, and no outputs were found seven times. Comparing NOTEARS and M-FRRMAB-HH on these 11 datasets, NOTEARS won three times in terms of the BIC score and only once in terms of the F1 score. This shows that the network learned by NOTEARS had a higher BIC score, and our algorithm was more likely to acquire a structure with lower structural error because of the addition of structural constraints. In our experiments, both BNC-PSO and the PCS adopted structural soft constraints and heuristic searches. However, for large-scale networks such as Munin, Andes and Pigs, the BIC scores and F1 scores both decreased significantly, which indicated that the heuristic methods they employ have poor search performance on large-scale networks, whereas our algorithm has greater global search capability and is suitable for structural learning on large-scale networks. The performance of PC-Stable and LiNGAM was significantly lower than that of the other score-based algorithms, and the performance varied dramatically on different datasets, especially on the Andes network, where the BIC score was three orders of magnitude different from that of the other algorithms.

On the basis of the above results and analysis, we can conclude that structural constraints improve the search efficiency and learning accuracy of our algorithm. Compared with the single heuristic algorithm, the multi-population hyper-heuristic algorithm has a greater global search ability and is a suitable solution for large DAG learning problems.

## 6. Conclusions and Future Research

In this work, soft constraints were obtained via partial correlation analysis, and the hard constraints were determined via expert knowledge. Both serve as structural constraints to guide our proposed hyper-heuristic algorithm to improve the search efficiency and accuracy of our algorithm. The effect of expert knowledge and LLH pool on the efficiency of our algorithm was discussed. The experimental results showed that M-FRRMAB-HH is an effective and accurate causal discovery method under linear SEMs. Compared to other state-of-the-art methods, it output structures that are closer to real causality. We still need to make more efforts to improve our method. In the LLH pool, the restart operator we designed has a high time cost, so we need to develop a better heuristic operator library.

## Figures and Tables

**Table 1:** Summary of networks.

Network	Nodes	Edges	Max.indeg	Max.outdeg	Avg.deg
Alarm	37	46	4	5	2.49
Hepar2	70	123	6	17	3.51
Win95pts	76	112	7	10	2.95
Munin	189	282	3	15	2.98
Andes	223	338	6	12	3.03
Pigs	441	592	2	39	2.68

**Table 2:** Parameters of the M-FRRMAB-HH.

Param.	Value	Description
*p*	0.1	The constraint rate of the edge that is sure to exist
*q*	0.5	The constraint rate of the nonexistent edge
*W*	10	the length of sliding window
*C*	10	Equilibrium coefficient
*D*	0.5	Decay coefficient
nPop	50	The population size
$L_{max}$	2×n	The maximum number of unpromoted iterations allowed
MaxIt	5000	The maximum number of iterations allowed
sn	5	The number of subgroups
μ	lnm	The threshold of pruning
$l_{m}$	20	Control parameters of the elimination and dispersal operator
zj	0.5	Percentage receiving expert guidance

**Table 3:** Performance of M-FRRMAB-HH algorithm on different datasets. Bold denotes that BIC is greater than SBS.

Network	SEM	BIC	SBS	AD	DD	RD	SET(s)	F1
alarm	1	$-1.8710\times 10^{4}$ ± 0	$-1.8710\times 10^{4}$	0 ± 0	0 ± 0	0 ± 0	1.58	1 ± 0
	2	$-1.8638\times 10^{4}$ ± 0	$-1.8638\times 10^{4}$	0 ± 0	0 ± 0	0 ± 0	1.27	1 ± 0
	3	$-6.3849\times 10^{3}$ ± 0	$-6.3849\times 10^{3}$	0 ± 0	0 ± 0	0 ± 0	1.17	1 ± 0
hepar2	1	$-3.5372\times 10^{4}$ ± 0	$-3.5372\times 10^{4}$	0 ± 0	0 ± 0	0 ± 0	20.40	1 ± 0
	2	$-3.5318\times 10^{4}$ ± 0	$-3.5318\times 10^{4}$	0 ± 0	0 ± 0	0 ± 0	20.57	1 ± 0
	3	$-1.2133\times 10^{4}$ ± 0	$-1.2133\times 10^{4}$	0 ± 0	0 ± 0	0 ± 0	15.31	1 ± 0
win95pts	1	$-3.8374\times 10^{4}$ ± 0	$-3.8374\times 10^{4}$	0 ± 0	0 ± 0	0 ± 0	13.28	1 ± 0
	2	**−3.8309 $\times \;10^{4}$** ± 0	$-3.8310\times 10^{4}$	0 ± 0	0 ± 0	1 ± 0	10.42	0.9911 ± 0
	3	$-1.3083\times 10^{4}$ ± 0	$-1.3083\times 10^{4}$	0 ± 0	0 ± 0	0 ± 0	12.91	1 ± 0
munin	1	**−9.5504 $\times \;10^{4}$** ± 0	$-9.5663\times 10^{4}$	0 ± 0	53 ± 0	0 ± 0	106.15	0.8963 ± 0
	2	**−9.5399 $\times \;10^{4}$** ± 0	$-9.5561\times 10^{4}$	0 ± 0	53 ± 0	1 ± 0	116.39	0.8924 ± 0
	3	**−3.2219 $\times \;10^{4}$** ± 0	$-3.2393\times 10^{4}$	0 ± 0	53 ± 0	0 ± 0	83.29	0.8963 ± 0
andes	1	$-1.1296\times 10^{5}$ ± 0	$-1.1296\times 10^{5}$	0 ± 0	0 ± 0	0 ± 0	345.71	1 ± 0
	2	**−1.1280 $\times \;10^{5}$** ± 0	$-1.1281\times 10^{5}$	1 ± 0	0 ± 0	0 ± 0	396.59	0.9985 ± 0
	3	$-3.8190\times 10^{4}$ ± 0	$-3.8190\times 10^{4}$	0 ± 0	0 ± 0	0 ± 0	402.05	1 ± 0
pigs	1	**−2.2207 $\times \;10^{5}$** ± 0	$-2.2301\times 10^{5}$	0 ± 0	324 ± 0	0 ± 0	1208.06	0.6233 ± 0
	2	**−2.2219 $\times \;10^{5}$** ± 0	$-2.2316\times 10^{5}$	1 ± 0	324 ± 0	0 ± 0	1484.07	0.6225 ± 0
	3	**−7.4305 $\times \;10^{4}$** ± 0	$-7.5375\times 10^{4}$	0 ± 0	324 ± 0	0 ± 0	1428.41	0.6233 ± 0

**Table 4:** Performance of M-FRRMAB-HH algorithm without hard constraints. Bold denotes that BIC is greater than SBS.

Network	SEM	BIC	SBS	AD	DD	RD	SET(s)	F1
alarm	1	$-1.8710\times 10^{4}$ ± 0	$-1.8710\times 10^{4}$	0 ± 0	0 ± 0	0 ± 0	2.29	1 ± 0
	2	$-1.8638\times 10^{4}$ ± 0	$-1.8638\times 10^{4}$	0 ± 0	0 ± 0	0 ± 0	2.14	1 ± 0
	3	$-6.3849\times 10^{3}$ ± 0	$-6.3849\times 10^{3}$	0 ± 0	0 ± 0	0 ± 0	1.95	1 ± 0
hepar2	1	$-3.5372\times 10^{4}$ ± 0	$-3.5372\times 10^{4}$	0 ± 0	0 ± 0	0 ± 0	28.07	1 ± 0
	2	$-3.5318\times 10^{4}$ ± 0	$-3.5318\times 10^{4}$	0 ± 0	0 ± 0	0 ± 0	24.57	1 ± 0
	3	$-1.2133\times 10^{4}$ ± 0	$-1.2133\times 10^{4}$	0 ± 0	0 ± 0	0 ± 0	23.25	1 ± 0
win95pts	1	$-3.8374\times 10^{4}$ ± 0	$-3.8374\times 10^{4}$	0 ± 0	0 ± 0	0 ± 0	19.16	1 ± 0
	2	**−3.8309 $\times \;10^{4}$** ± 0	$-3.8310\times 10^{4}$	0 ± 0	0 ± 0	1 ± 0	14.12	0.9911 ± 0
	3	$-1.3083\times 10^{4}$ ± 0	$-1.3083\times 10^{4}$	0 ± 0	0 ± 0	0 ± 0	18.61	1 ± 0
munin	1	**−9.5488 $\times \;10^{4}$** ± 0	$-9.5663\times 10^{4}$	0 ± 0	59 ± 0	0 ± 0	160.46	0.8832 ± 0
	2	**−9.5380 $\times \;10^{4}$** ± 0	$-9.5561\times 10^{4}$	0 ± 0	59 ± 0	1 ± 0	179.94	0.8792 ± 0
	3	**−3.2199 $\times \;10^{4}$** ± 0	$-3.2393\times 10^{4}$	0 ± 0	59 ± 0	0 ± 0	177.87	0.8832 ± 0
andes	1	$-1.1296\times 10^{5}$ ± 0	$-1.1296\times 10^{5}$	0 ± 0	0 ± 0	0 ± 0	660.71	1 ± 0
	2	**−1.1280 $\times \;10^{5}$** ± 0	$-1.1281\times 10^{5}$	1 ± 0	0 ± 0	0 ± 0	559.27	0.9985 ± 0
	3	$-3.8190\times 10^{4}$ ± 0	$-3.8190\times 10^{4}$	0 ± 0	0 ± 0	0 ± 0	676.99	1 ± 0
pigs	1	**−2.2197 $\times \;10^{5}$** ± 0	$-2.2301\times 10^{5}$	0 ± 0	357 ± 0	0 ± 0	2850.50	0.5683 ± 0
	2	**−2.2209 $\times \;10^{5}$** ± 0	$-2.2316\times 10^{5}$	1 ± 0	357 ± 0	0 ± 0	3184.71	0.5676 ± 0
	3	**−7.4199 $\times \;10^{4}$** ± 0	$-7.5375\times 10^{4}$	0 ± 0	357 ± 0	0 ± 0	2984.92	0.5683 ± 0

**Table 5:** Performance of M-FRRMAB-HH algorithm under different LLHs.

Network	LLH	BIC	AD	DD	RD	SET(s)	F1
alarm	1	$-1.8710\times 10^{4}$ ± 0	0 ± 0	0 ± 0	0 ± 0	1.78	1 ± 0
	2	$-1.8710\times 10^{4}$ ± 0	0 ± 0	0 ± 0	0 ± 0	1.53	1 ± 0
	3	$-1.8710\times 10^{4}$ ± 0	0 ± 0	0 ± 0	0 ± 0	1.45	1 ± 0
hepar2	1	$-3.5733\times 10^{4}$ ± 311.46	0 ± 0	2 ± 0.71	0 ± 0	22.62	0.9918 ± 0.0029
	2	$-3.7564\times 10^{4}$ ± 1225.67	0 ± 0	1.60 ± 0.89	0 ± 0	13.30	0.9934 ± 0.0037
	3	$-4.9708\times 10^{4}$ ± 0	0 ± 0	12 ± 0	0 ± 0	7.31	0.9487 ± 0
win95pts	1	$-3.8496\times 10^{4}$ ± 35.49	0.80 ± 1.10	1 ± 0	0 ± 0	15.70	0.9920 ± 0.0048
	2	−3.8440 $\times \;10^{4}$ ± 146.06	0 ± 0	0.20 ± 0.45	0 ± 0	12.21	0.9991 ± 0.0020
	3	$-4.0845\times 10^{4}$ ± 35.49	0.80 ± 1.10	3 ± 0	0 ± 0	7.40	0.9829 ± 0.0048
munin	1	−9.5504$\;\times \;10^{4}$ ± 0	0 ± 0	53 ± 0	0 ± 0	502.06	0.8963 ± 0
	2	−9.5504$\;\times \;10^{4}$ ± 0	0 ± 0	53 ± 0	0 ± 0	71.03	0.8963 ± 0
	3	−9.7568$\;\times \;10^{4}$ ± 0	0 ± 0	57 ± 0	0 ± 0	44.17	0.8876 ± 0
andes	1	$-1.1296\;\times \;10^{5}$ ± 0	0 ± 0	0 ± 0	0 ± 0	785.12	1 ± 0
	2	−1.3746$\;\times \;10^{5}$ ± $3.3554\;\times \;10^{4}$	1.60 ± 2.19	2 ± 2.74	0 ± 0	574.69	0.9947 ± 0.0073
	3	$-3.2952\times 10^{6}$ ± $4.2327\times 10^{6}$	8.80 ± 0.45	13.40 ± 0.55	2.40 ± 0.55	303.37	0.9598 ± 0.0021
pigs	1	−2.2207$\;\times \;10^{5}$ ± 0	0 ± 0	324 ± 0	0 ± 0	785.58	0.6233 ± 0
	2	−2.2207$\;\times \;10^{5}$ ± 0	0 ± 0	324 ± 0	0 ± 0	779.83	0.6233 ± 0
	3	−2.2207$\;\times \;10^{5}$ ± 0	0 ± 0	324 ± 0	0 ± 0	194.52	0.6233 ± 0

**Table 6:** F1 scores of the algorithms for different SEMs. Bold denotes the F1 score that was the best amongst all methods. “-” indicates that no result was displayed.

SEM	Network	PC-Stable	LiNGAM	PCS	NOTEARS	BNC-PSO	M-FRRMAB-HH
SEM1	alarm	0.8539	0.6387	0.9787	0.9892	0.9892	**1**
	hepar2	0.5871	0.7103	0.8593	-	0.9049	**1**
	win95pts	0.7882	0.4683	0.8631	0.9912	0.9715	**1**
	munin	0.7546	0.3350	0.6314	-	0.8611	**0.8832**
	andes	0.5497	0.2823	0.6826	-	0.8768	**1**
	pigs	0.4766	0.1650	0.3612	0.5683	0.5272	**0.5683**
SEM2	alarm	0.8605	0.6116	0.9787	0.9451	0.9778	**1**
	hepar2	0.6346	0.7117	0.8651	0.7603	0.9048	**1**
	win95pts	0.8491	0.6154	0.9136	0.9646	0.9695	**0.9911**
	munin	0.7976	0.3724	0.6216	0.8151	0.8607	**0.8792**
	andes	0.8394	0.4008	0.6000	-	0.9327	**0.9985**
	pigs	0.4174	0.1499	0.3265	0.5600	0.5141	**0.5676**
SEM3	alarm	0.8276	0.9388	0.9892	0.9053	**1**	**1**
	hepar2	0.5545	0.7413	0.8696	-	0.9392	**1**
	win95pts	0.8020	0.5542	0.9912	0.9442	0.9713	**1**
	munin	0.7670	0.3627	0.7876	-	0.8739	**0.8832**
	andes	0.5209	0.2649	0.7862	-	0.9200	**1**
	pigs	0.4687	0.1783	0.5098	0.5642	0.5631	**0.5683**

**Table 7:** BIC scores of the algorithms for different SEMs. Bold denotes the BIC score that was the best amongst all methods. “-” indicates that no result was displayed.

SEM	Network	PC-Stable	LiNGAM	PCS	NOTEARS	BNC-PSO	M-FRRMAB-HH
SEM1	alarm	$-2.23\times 10^{4}$	$-2.15\times 10^{4}$	$-1.87\times 10^{4}$	$-1.87\times 10^{4}$	−1.87 $\times 10^{4}$	**−1.87$\;\times \;10^{4}$**
	hepar2	$-1.48\times 10^{5}$	$-4.26\times 10^{4}$	$-3.97\times 10^{4}$	-	$-4.73\times 10^{4}$	**−3.54$\;\times \;10^{4}$**
	win95pts	$-7.70\times 10^{4}$	$-4.94\times 10^{4}$	$-4.09\times 10^{4}$	$-3.84\times 10^{4}$	$-4.08\times 10^{4}$	**−3.84$\;\times \;10^{4}$**
	munin	$-1.29\times 10^{5}$	$-1.51\times 10^{5}$	$-1.22\times 10^{5}$	-	$-9.86\times 10^{4}$	**−9.55$\;\times \;10^{4}$**
	andes	$-3.97\times 10^{8}$	$-5.77\times 10^{8}$	$-4.39\times 10^{5}$	-	$-6.49\times 10^{6}$	**−1.13$\;\times \;10^{5}$**
	pigs	$-2.28\times 10^{5}$	$-2.46\times 10^{5}$	$-2.26\times 10^{5}$	**−2.22 $\times \;10^{5}$**	$-2.23\times 10^{5}$	−2.22$\;\times \;10^{5}$
SEM2	alarm	$-2.04\times 10^{4}$	$-1.95\times 10^{4}$	$-1.86\times 10^{4}$	$-1.88\times 10^{4}$	$-1.90\times 10^{4}$	**−1.86$\;\times \;10^{4}$**
	hepar2	$-4.48\times 10^{4}$	$-3.79\times 10^{4}$	$-3.63\times 10^{4}$	$-3.68\times 10^{4}$	$-3.69\times 10^{4}$	**−3.53$\;\times \;10^{4}$**
	win95pts	$-4.63\times 10^{4}$	$-4.03\times 10^{4}$	$-3.83\times 10^{4}$	$-3.84\times 10^{4}$	$-3.85\times 10^{4}$	**−3.83$\;\times \;10^{4}$**
	munin	$-9.70\times 10^{4}$	$-1.06\times 10^{5}$	$-9.85\times 10^{4}$	$-9.59\times 10^{4}$	$-9.57\times 10^{4}$	**−9.54$\;\times \;10^{4}$**
	andes	$-1.52\times 10^{5}$	$-1.47\times 10^{5}$	$-1.24\times 10^{5}$	-	$-1.18\times 10^{5}$	**−1.13$\;\times \;10^{5}$**
	pigs	$-2.25\times 10^{5}$	$-2.32\times 10^{5}$	$-2.23\times 10^{5}$	**−2.22 $\times \;10^{5}$**	$-2.22\times 10^{5}$	−2.22$\;\times \;10^{5}$
SEM3	alarm	$-7.87\times 10^{3}$	$-6.40\times 10^{3}$	$-6.39\times 10^{3}$	$-6.50\times 10^{3}$	**−6.38 $\times \;10^{3}$**	**−6.38$\;\times \;10^{3}$**
	hepar2	$-3.90\times 10^{4}$	$-1.57\times 10^{4}$	$-1.38\times 10^{4}$	-	$-1.69\times 10^{4}$	**−1.21$\;\times \;10^{4}$**
	win95pts	$-2.07\times 10^{4}$	$-1.61\times 10^{4}$	$-1.31\times 10^{4}$	$-1.32\times 10^{4}$	$-1.39\times 10^{4}$	**−1.31$\;\times \;10^{4}$**
	munin	$-4.68\times 10^{4}$	$-4.28\times 10^{4}$	$-3.37\times 10^{4}$	-	$-3.25\times 10^{4}$	**−3.22$\;\times \;10^{4}$**
	andes	$-1.88\times 10^{8}$	$-6.22\times 10^{7}$	$-1.04\times 10^{5}$	-	$-2.35\times 10^{6}$	**−3.82$\;\times \;10^{4}$**
	pigs	$-7.60\times 10^{4}$	$-8.31\times 10^{4}$	$-7.55\times 10^{4}$	**−7.42$\;\times \;10^{4}$**	$-7.44\;\times 10^{4}$	−7.43$\;\times \;10^{4}$

## Data Availability

The true networks of all eight datasets are known, and they are publicly available (http://www.bnlearn.com/bnrepository, accessed on 30 October 2024).

## Abbreviations

The following abbreviations are used in this manuscript:

ABC	Artificial bee colony
BFO	Bacterial foraging optimization
BIC	Bayesian information criterion
CI	Conditional independence
CSS	Complete search space
DAG	Directed acyclic graph
FIR	fitness improvement rate
FRRMAB	Fitness-rate-rank-based multiarmed bandit
GSS	Global search space
LLH	Low-level heuristic
LSS	Local search space
MB	Markov blanket
NLL	Negative log-likelihood
PSO	Particle swarm optimization
SEM	Structural equation model
SPPC	Structural priors by partial correlation
